# Diagnostic accuracy of mutational analysis along the Müllerian tract to detect ovarian cancer

**DOI:** 10.1136/ijgc-2022-003911

**Published:** 2022-11-16

**Authors:** Majke H.D. van Bommel, Johanna M.A. Pijnenborg, Louis J M van der Putten, Johan Bulten, Marc P.L.M. Snijders, Heidi V.N. Küsters-Vandevelde, Sanne Sweegers, M. Caroline Vos, Marjolein J.L. Ligtenberg, Astrid Eijkelenboom, Joanne A de Hullu, Casper Reijnen

**Affiliations:** 1 Obstetrics & Gynaecology, Radboud Institute for Health Science, Radboudumc, Nijmegen, The Netherlands; 2 Pathology, Radboudumc, Nijmegen, The Netherlands; 3 Obstetrics and Gynaecology, Catharina Hospital, Eindhoven, The Netherlands; 4 Pathology, Catharina Hospital, Eindhoven, The Netherlands; 5 Obstetrics and Gynaecology, Elisabeth-TweeSteden Ziekenhuis, Tilburg, The Netherlands; 6 Human Genetics, Radboudumc, Nijmegen, The Netherlands; 7 Radiation Oncology, Radboudumc, Nijmegen, The Netherlands

**Keywords:** Ovarian Cancer, Pathology

## Abstract

**Objective:**

Ovarian cancer is known for its poor prognosis, which is mainly due to the lack of early symptoms and adequate screening options. In this study we evaluated whether mutational analysis in cervicovaginal and endometrial samples could assist in the detection of ovarian cancer.

**Methods:**

In this prospective multicenter study, we included patients surgically treated for either (suspicion of) ovarian cancer or for a benign gynecological condition (control group). A cervicovaginal self-sample, a Papanicolaou (Pap) smear, a pipelle endometrial biopsy, and the surgical specimen were analyzed for (potentially) pathogenic variants in eight genes (*ARID1A*, *CTNNB1*, *KRAS*, *MTOR*, *PIK3CA*, *POLE*, *PTEN*, and *TP53*) using single-molecule molecular inversion probes. Sensitivity and specificity were calculated to assess diagnostic accuracy.

**Results:**

Based on surgical histology, our dataset comprised 29 patients with ovarian cancer and 32 controls. In 83% of the patients with ovarian cancer, somatic (potentially) pathogenic variants could be detected in the final surgical specimen, of which 71% included at least a *TP53* variant. In 52% of the ovarian cancer patients, such variants could be detected in either the self-sample, Pap smear, or pipelle. The Pap smear yielded the highest diagnostic accuracy with 26% sensitivity (95% CI 10% to 48%). Overall diagnostic accuracy was low and was not improved when including *TP53* variants only.

**Conclusions:**

Mutational analysis in cervicovaginal and endometrial samples has limited accuracy in the detection of ovarian cancer. Future research with cytologic samples analyzed on methylation status or the vaginal microbiome may be relevant.

**What is already known on this topic**

- DNA variants related to ovarian cancer can be detected along the Müllerian tract. In this study we compared DNA pathogenic variants in cervicovaginal self-samples, Papanicolaou (Pap) smears, and pipelle endometrial biopsies with the pathogenic variants in the surgical specimen in patients with ovarian cancer and control patients. We assessed diagnostic accuracy of detecting ovarian cancer with those samples.

**What this study adds**

- We found that diagnostic accuracy was low for cervicovaginal self-samples, Pap smears, and endometrial biopsies. Thus, the samples assessed in this way cannot be used for early detection of ovarian cancer.

**How this study might affect research, practice, or policy**

- This study contributes to further unraveling the oncogenesis of ovarian carcinoma and assists in research regarding the urgently needed detection of ovarian cancer.

## Introduction

Epithelial ovarian cancer is the most lethal gynecological cancer.^1^ Patients generally present with advanced-stage disease leading to a 5-year survival of approximately 45%,^2^ mainly due to the absence of early symptoms and reliable screening methods.^3^ By contrast, survival for the limited number of patients with localized disease is around 92%, suggesting that early detection of epithelial ovarian cancer could substantially improve prognosis.^2^

Epithelial ovarian cancer is thought to develop from tissues embryologically derived from the Müllerian ducts (fallopian tubes, uterus, upper part of the vagina) with the ovaries secondarily involved. Nowadays, ovarian, fallopian tubal, and/or the peritoneal malignancies are considered collectively as ovarian carcinomas, of which approximately 75% are high-grade serous carcinomas. There is compelling evidence that high-grade serous carcinoma originates in the fallopian tubes,^4^ potentially offering new strategies for ovarian cancer prevention and early detection.

Screening for ovarian cancer using transvaginal sonography and cancer antigen 125 (CA125) has been proven ineffective in the general population^3^ and in women at increased inherited risk.^5^ Lately, instead of focusing on macroscopic changes, there is increasing interest in detecting microscopic (pre)malignant cells that detach along the Müllerian ducts. Interest in DNA analysis in cytological samples is growing since (cell-free) DNA variants can be detected in cytological samples even without the presence of tumor cells. Kinde et al extracted DNA from a Papanicolaou (Pap) smear and found a sensitivity of detecting ovarian cancer of 41%, particularly driven by mutated Tumor Protein 53 (*TP53*) variants.^6^ When combining mutational analysis with DNA extracted from a Pap smear with plasma, sensitivity improved to 63%. An intra-uterine brush to sample DNA closer to the primary source increased sensitivity.^7^ Currently, uterine and tubal lavage to detect early-stage ovarian cancer is being investigated (NCT 02039388). The first results are promising as ovarian cancer cells could be collected in 24 of 30 patients with ovarian cancer, and mainly *TP53* mutations could be identified.^8^

The above findings support the presence of ovarian cancer cells along the Müllerian tract, which could potentially be detected with minimally invasive sampling methods. Therefore, we investigated the diagnostic accuracy of detecting ovarian cancer by assessing DNA pathogenic variants in cervicovaginal and endometrial samples and comparing them with the pathogenic variants found in the tumor itself.

## Methods

### Design and Population

This prospective observational multicenter study included consecutive patients undergoing surgery for high suspicion of ovarian cancer or for a benign gynecological condition (control group) in three Dutch hospitals: Radboud University Medical Center, Nijmegen; Canisius-Wilhelmina Hospital, Nijmegen; and Elisabeth-TweeSteden Hospital, Tilburg. Suspicion was based on a Risk Malignancy Index >200, the presence of ascites, peritoneal depositions, omental cake, or laparoscopic evaluation. Inclusion criteria were adult age and surgery between December 2013 and January 2017 in a participating hospital. Exclusion criteria were a history of pelvic radiotherapy or previous hysterectomy. Ethical approval was obtained in all hospitals (Study Number 2013/451) and each patient signed informed consent. The study was prospectively registered at the Dutch Trial Registry (NTR4299) and performed according to the STARD guidelines for Standards for the Reporting of Diagnostic accuracy studies. Patients with endometrial cancer were included as well. Their results have been published previously.^9^

### Data Collection

**Figure 1:**
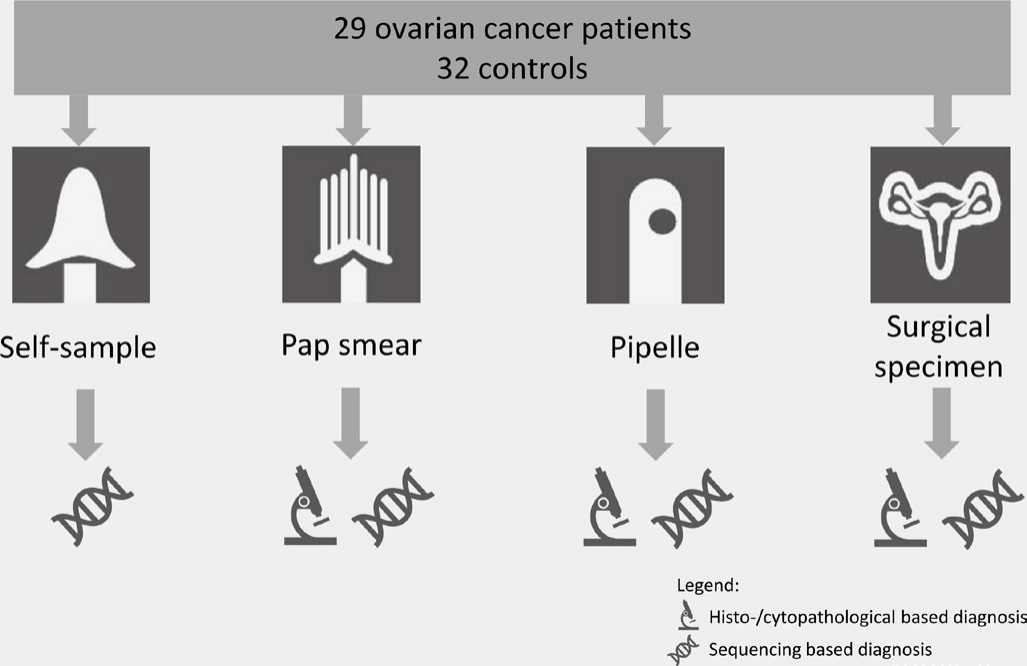
Study flowchart.

Four specimens were collected from each patient: cervicovaginal self-sample, Pap smear, pipelle endometrial biopsy, and surgical sample (ovarian tissue) (Figure 1). Samples were collected in the aforementioned order by the operating gynecologist on the day of surgery. Demographic information was extracted from medical records.

### Pathogenic Variant Analysis

The complete workflow is provided in Online supplemental document 1. Briefly, DNA was extracted from the four specimens and analyzed using single-molecule molecular inversion probes-based sequencing on a NextSeq500 device (Illumina, San Diego, California, USA), as previously described.^10^ The single-molecule molecular inversion probes were constructed to highlight hotspots in the oncogenes relevant in ovarian and endometrial cancer: Catenin Beta 1 (*CTNNB1),* Kirsten rat sarcoma virus (*KRAS),* mammalian target of rapamycin (*MTOR),* Phosphatidylinositol-4,5-Bisphosphate 3-Kinase Catalytic Subunit Alpha (*PIK3CA),* and Polymerase ε (*POLE*); and all coding and splice site sequences of the tumor suppressor genes: AT-rich interactive domain-containing protein 1A (*ARID1A*), Phosphatase and tensin homolog (*PTEN),* and Tumor Protein 53 (*TP53*). Genes were chosen based on the genetic characteristics of ovarian and endometrial cancer as described in The Cancer Genome Atlas (TCGA).^11–13^ Variants were categorized according to the following classes: 1, benign; 2, likely benign; 3, variant of unknown significance; 4, likely pathogenic, and 5, pathogenic. The last three classes were considered (potentially) pathogenic.

10.1136/ijgc-2022-003911.supp1 Supplementary data

All surgical ovarian samples and pipelle endometrial biopsies were analyzed for the presence of the (potentially) pathogenic variants with a variant allele frequency of ≥3% and a minimal number of five variant reads (equal to three unique genomic DNA molecules). Additionally, the pipelle data were evaluated at the positions with known pathogenic variants in the surgical specimen with a cut-off of five unique variant reads and no minimal variant allele frequency. For evaluation of the Pap smears and self-samples, two independent library preparations were analyzed using a minimal variant allele frequency of 1% as we expected low variant allele frequencies with the samples mainly containing healthy endocervical cells and few tumor cells.

### Data Analysis

Baseline data were analyzed descriptively and differences between groups were analyzed using a t-test, Mann–Whitney U test, or χ^2^ test. To measure diagnostic accuracy, we calculated sensitivity and specificity. First, we calculated the detection rates of (potentially) pathogenic variants per sample among all patients. Second, we measured diagnostic accuracy among all patients using the detection of (potentially) pathogenic variants in their surgical sample as gold standard. Third, we focused on *TP53* pathogenic variants as these variants are primarily related to ovarian cancer.^14^

## Results

Specimens were collected from 37 patients with ovarian cancer and 32 controls. Eight patients with ovarian cancer were excluded because sequencing of the surgical specimen (the gold standard) was unsuccessful: the coverage was too low to detect any potential variant. Thus, 29 patients with ovarian cancer and 32 controls were included. Table 1 shows the characteristics of the patients.

**Table 1:** Baseline characteristics

	Ovarian cancer patients (n=29)	Controls (n=32)	P value
Age, years	66 (32–83)	57 (45–82)	0.063
Body mass index, kg/m^2^	25 (19–32)	23 (20–44)	0.644
CA125, kIU/L	360 (8–2196)	20 (9–142)	<0.001
Menopausal status			0.321
Pre-menopausal	4 (14)	8 (25)	
Post-menopausal	25 (86)	23 (72)	
Unknown	0	1 (3)	
Histology			
High-grade serous	20 (69)		
Endometrioid	3 (10)		
Clear cell	1 (3)		
Malignant mixed Müllerian tumor	1 (3)		
Mixed*	4 (14)		
Myoma		9 (28)	
Cystadenoma ovarii		10 (31)	
Fibroma/teratoma ovarii		7 (22)	
Other†		6 (19)	
FIGO stage		NA	
IA	1 (3)		
IB	0		
IC	2 (7)		
IIA	2 (7)		
IIB	2 (7)		
IIC	0		
IIIA	1 (3)		
IIIB	4 (14)		
IIIC	14 (48)		
IV	3 (10)		

Values presented as median (range) or N (%).

*Mixed histology included two clear cell/serous, one clear cell/endometrioid, and one serous/endometrioid.

†Other histology included one adenomyosis, one inflammation, two prolapse, and two normal.

CA125, cancer antigen 125; FIGO, International Federation of Gynecology and Obstetrics.

### Surgical Specimens

**Figure 2:**
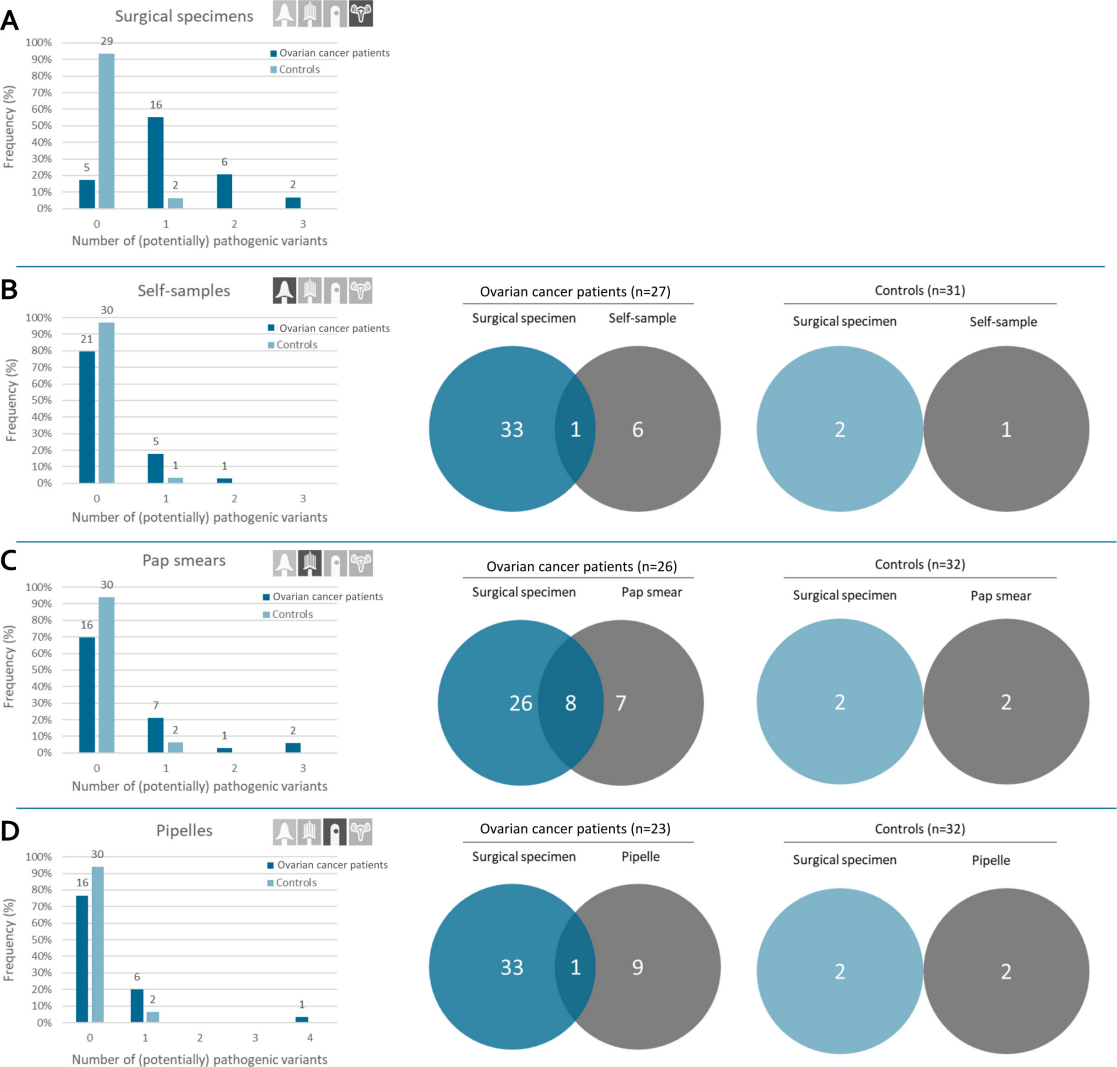
Overview of detected (potentially) pathogenic variants in the surgical specimens (A), self-samples (B), Pap smears (C), and pipelle endometrial biopsies (D) and visualization of overlapping and non-overlapping variants between the surgical specimen and self-samples (B), Pap smears (C), and pipelle (D) in patients with ovarian cancer and controls.

In the tumors of 29 patients with ovarian cancers, 79% of the exons had a mean coverage of >250 reads, reflecting a 95% probability of detecting a variant, ‘adequately sequenced’ (see Online supplemental table 2).^10^ We detected at least one pathogenic variant in 24 patients (83%). In total, 34 (potentially) pathogenic variants were detected (Figure 2A). Among the 24 patients with ovarian cancer with pathogenic variants, 17 had a *TP53* variant (71%). Fifteen of these 17 had high-grade serous carcinoma, one had a clear cell/endometrioid ovarian cancer, and one a clear cell/serous ovarian cancer. Other detected variants were: *PIK3CA* (21%), *CTNNB1* (14%), *PTEN* (10%), *KRAS* (7%), *ARID1A* (3%), and *MTOR* (3%) (Table 2). Of the five patients without a detected variant in their surgical sample (17%), three had high-grade serous carcinoma, one had a clear cell carcinoma, and one had clear cell/serous histology. Among the controls, two patients (6%), both with a mucinous cystadenoma, were found to have a *KRAS* variant in their surgical specimen. Individual characteristics are shown in Online supplemental table 3.

**Table 2:** Number of (potentially) pathogenic variants in the various specimens

Ovarian cancer patients (n=29)				
(**Potentially) pathogenic variant**	**Self-sample** 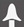 (**n=27**)	**Pap smear** 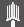 (**n=26**)	**Pipelle** 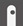 (**n=23**)	**Surgical sample** 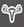 (**n=29**)
*TP53*	2 (7)	6 (19)	2 (9)	17 (59)
*PIK3CA*	3 (11)	5 (15)	4 (17)	6 (21)
*CTNNB1*	0	1 (4)	0	4 (14)
*PTEN*	0	1 (4)	1 (4)	3 (10)
*KRAS*	0	1 (4)	2 (9)	2 (7)
*ARID1A*	2 (7)	1 (4)	1 (4)	1 (3)
*MTOR*	0	0	0	1 (3)
*POLE*	0	0	0	0
Total number of pathogenic variants	7	15	10	34
Patients without a pathogenic variant	21 (78)	16 (62)	16 (70)	5 (17)
**Control patients (n=32**)				
(**Potentially) pathogenic variant**	**Self-sample** 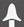 (**n=31**)	**Pap smear** 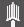 (**n=32**)	**Pipelle** 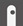 (**n=32**)	**Surgical sample** 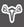 (**n=31**)
*TP53*	0	0	0	0
*PIK3CA*	0	2 (6)	2 (6)	0
*CTNNB1*	0	0	0	0
*PTEN*	0	0	0	0
*KRAS*	0	0	0	2 (6)
*ARID1A*	1 (3)	0	0	0
*MTOR*	0	0	0	0
*POLE*	0	0	0	0
Total number of pathogenic variants				
Patients without a pathogenic variant	30 (97)	30 (94)	30 (94)	29 (94)

Values are presented as number of pathogenic variants (% of patients).

Percentages may total >100% as one patient can have multiple variants.

### Cervicovaginal Samples (Self-Samples and Pap Smears)

Analysis of the cervicovaginal self-samples of 27 patients with ovarian cancer identified six patients (22%) with a total of seven (potentially) pathogenic variants: *PIK3CA* (n=3), *TP53* (n=2), and *ARID1A* (n=2); 98% of the exons were ‘adequately sequenced’. When evaluating overlapping variants (variants that were detected in more than one specimen), we found that two variants were found in all samples but the surgical specimen (one *TP53* variant, one *PIK3CA*); and one variant was found in the surgical specimen, self-sample and pipelle but not in the Pap smear (*ARID1A*). Four variants were solely found in the self-sample (see Figure 2B and Online supplemental table 4). One control had an *ARID1A* variant in her self-sample that was not detected in her other specimens.

Results of the Pap smears were available for 26 patients with ovarian cancer; exons ‘adequately sequenced’: 98%. Among them, 10 patients (38%) had one or more (potentially) pathogenic variants. In total, we detected 15 variants of which eight were overlapping with the variants in the surgical specimen (53%) (see Figure 2C and Online supplemental table 4). The eight overlapping variants were detected in six patients. Four of these six patients had high-grade serous carcinoma (of which three had a *TP53* variant (two stage 3C and one stage 2A) and one had a *PIK3CA*, *KRAS* and *PTEN* variant (high-grade serous carcinoma stage 3C); one had an endometrioid carcinoma stage 2A (and *CTNNB1* variant); and one had a clear cell/endometrioid carcinoma stage 1C (and *PIK3CA* variant)). Two control patients had both a *PIK3CA* variant solely in their Pap smear.

### Pipelle Endometrial Biopsies

A pipelle was available for 23 patients with ovarian cancer; 71% of the exons were ‘adequately sequenced’. Analysis showed seven patients (30%) with a total of 10 pathogenic variants: four *PIK3CA*, two *TP53*, two *KRAS*, one *PTEN*, and one *ARID1A*. We found one overlapping variant between the surgical specimens and the pipelles (10%), which was an *ARID1A* variant in a woman with high-grade serous carcinoma stage 3B who had this variant in all specimens but the Pap smear (see Figure 2D and Online supplemental table 4). Two control patients (6%) had a *PIK3CA* variant which were not found in their other specimens.

### Diagnostic Accuracy

Among the 29 patients with ovarian cancer, at least one (potentially) pathogenic variant was detected in 83% of the surgical specimens, in 22% of the self-samples, in 38% of the Pap smears, and in 30% of the pipelles. In the patients with ovarian cancer the detection rate for a (potentially) pathogenic variant in any of the sampling methods (the self-sample, Pap smear, or pipelle) was 52%. Among the controls, a false positive variant was detected in 6%, 3%, 6%, and 6%, respectively, of the specimens. Detection rates were roughly similar between early and late stage (see Supplementary Document 2). No correlation was found between the variant allele frequency in the ovarian tumor and the likelihood of detecting the variant in any of the sampling methods (data not shown).

The diagnostic accuracy of overlapping (potentially) pathogenic variants—for example, a pathogenic variant in minimally one of the sampling methods among patients with a pathogenic variant in their surgical specimen (n=24) and controls without a pathogenic variant in the surgical specimen (n=30)—is shown in Table 3. Sensitivity and specificity for an overlapping pathogenic variant in the surgical specimen and any of the sampling methods were 29% (95% CI 13% to 51%) and 87% (95% CI 69% to 96%), respectively.

**Table 3:** Diagnostic accuracy of various combinations of measurements to detect ovarian cancer

	Sensitivity	Specificity
Any (potentially) pathogenic variant (all patients)		
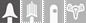	22 (9 to 43)	97 (83 to 100)
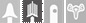	38 (20 to 59)	94 (79 to 99)
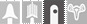	30 (13 to 53)	94 (79 to 99)
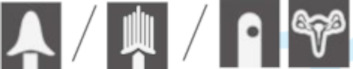	52 (33 to 71)	88 (71 to 96)
(Potentially) pathogenic variants overlapping with the surgical specimen		
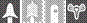	5 (0 to 23)	97 (83 to 100)
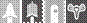	26 (10 to 48)	93 (80 to 99)
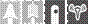	6 (0 to 27)	93 (80 to 99)
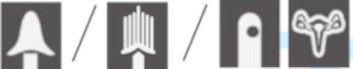	29 (13 to 51)	87 (69 to 96)

Diagnostic accuracy includes sensitivity and specificity.

Values are presented as percentage (95% CI)

When analyzing *TP53* pathogenic variants only, we identified 21 patients with ovarian cancer with 22 *TP53* variants, of whom 17 had the *TP53* variant in their surgical specimen. Overlapping *TP53* variants with the surgical specimen were found in the Pap smear in three patients (sensitivity 18% (95% CI 4% to 43%)). No overlapping *TP53* variants were found in the self-samples or the pipelles. Among controls, no *TP53* variants were detected (100% specificity).

## Discussion

### Summary of Main Results

In this multicenter prospective study we investigated the diagnostic accuracy of detecting ovarian cancer with mutational analysis in cervicovaginal and endometrial biopsies. We found (potentially) pathogenic variants in 83% of the ovarian cancer tumors, of which 71% were *TP53* variants. In 52%, a (potentially) pathogenic variant could be detected in either a cervicovaginal self-sample, Pap smear, or pipelle. Sensitivity was low for all sampling methods and remained low when analyzing *TP53* variants only. Among the controls, hardly any variants were detected, resulting in very high specificity of all sampling methods.

### Results in the Context of Published Literature

Despite an impressive research effort to improve the therapeutic options for ovarian cancer, the survival rate has barely increased over the past decades.^2^ Diagnosing epithelial ovarian cancer in an early stage might improve prognosis substantially, emphasizing the need for early detection methods.^2^ It is notable that early stage detection seemed not to be inferior to late stage detection in our study. Based on anatomical position, one could reason that pathogenic variants would be more frequently found in endometrial biopsies compared with cervicovaginal samples. Some studies reported on potential precursors of serous epithelial ovarian cancer in the endometrium,^15 16^ although the fallopian tubes are nowadays considered as the site of origin of mainly serous epithelial ovarian cancer.^4^ Thus far, no research has been published about sampling the endometrium with a pipelle biopsy to potentially detect ovarian cancer early. However, as we found, mutational analysis of the endometrium obtained via a pipelle seems not to be appropriate for this purpose, although this does not exclude the potential role of the uterus and/or endometrium in early ovarian cancer detection. The lower prevalence of diagnosed pathogenic variants in the pipelle biopsies compared with the cervicovaginal samples could be explained by the fact that endometrial tissue was processed in paraffin, in which DNA preservation is less optimal leading to a lower sequence coverage and thus lower sensitivity. The cervicovaginal samples were stored in PreservCyt medium, which better maintains DNA stability.^17^ Future studies could investigate whether the detection rate of pathogenic variants would increase when analyzing DNA from pipelle samples being preserved in PreservCyt medium.

As demonstrated, cytology samples might be more promising in early ovarian cancer detection than endometrial histology samples. This is especially attractive as obtaining cervical cytology samples is highly accepted and less invasive than obtaining endometrial histology. Of the 34 detected variants in the surgical specimens, eight were also found in the Pap smear (24%) whereas we only detected one variant in the cytological cervicovaginal self-sample overlapping with the surgical specimen (3%). Our results are very similar to those of Wang et al,^7^ who found that 29% of patients with ovarian cancer harbored detectable variants, mostly *TP53*, in their Pap smears. They also investigated intra-uterine cytology sampling using Tao brushes which could detect a variant in 42% of patients with ovarian cancer. Current research is investigating whether uterine cytology samples, obtained via lavage of the uterine cavity and analyzed with next-generation sequencing, can serve as an early detection method (NCT 02039388). Combining the results of their uterine cytology samples with cytologic assessed pipelle biopsies might show a new insight into the etiology of ovarian cancer. Also, the methylation status of such cytologic samples may contribute/improve ovarian cancer detection. Moreover, Barrett et al recently demonstrated that the DNA methylome in cervical samples can predict the risk of ovarian cancer with about 75% certainty.^18^ Evaluation of the vaginal microbiome as a possible early detection method could also be promising. The microbiome might impact estrogen metabolism and may influence the risk of ovarian cancer, like exogenous estrogens.^19–22^ In colorectal oncogenesis the microbiota seem to play a major role,^23^ which may also apply to gynecological cancers.

Considering the oncogenesis of high-grade serous carcinoma, we expected to find more *TP53* pathogenic variants. Moreover, approximately 90% of all patients with serous epithelial ovarian cancer have a *TP53* variant.^14^ We found a *TP53* variant in 59% of patients, which might be explained by tumor heterogeneity.^24^ Also, three of our five patients without a pathogenic variant in the surgical specimen had a high-grade serous carcinoma. Further, *ARID1A* variants were under-represented among patients with clear cell histology based on TCGA, probably because only four patients had (mixed) clear cell histology. Our detection rate may have been higher if we had included genes involved in homologous recombination as these are commonly related with epithelial ovarian cancer. For example, somatic *BReast CAncer* pathogenic variants can be detected in about 17% of all patients with epithelial ovarian cancer.^25^

### Strengths and Weaknesses

Our study is the first to sample the endometrium with a pipelle to potentially detect ovarian cancer. We covered most of the Müllerian tract by sampling the endometrium, cervix, and vagina, in addition to the tumor. There are, however, some limitations. A larger sample size would strengthen our results. The low prevalence of (potentially) pathogenic variants overall might be related to insufficiently deep sequencing of some samples, the choice of the library preparation method, and the content of the gene panel, although we expected that the majority would be picked up with this panel. The detection rate may have been higher when the pipelle samples were stored in PreservCyt medium. There might be some false positive variants as healthy persons appear sometimes to have pathogenic variants as well.^26 27^ Furthermore, the class III pathogenic variants were considered (potentially) pathogenic, although these reflect a minority of all variants. Thus far it is unknown whether or not these variants should be considered pathogenic.

### Implications for Practice and Future Research

This study contributes to the unraveling of ovarian cancer etiology and assists in research regarding the urgently needed detection of ovarian cancer. For future research it would be relevant to investigate cytology samples acquired along the Müllerian tract, stored in PreservCyt medium, using alternative library preparation methods, expanding the gene panel, and potentially analyzing the methylation status or the vaginal microbiome.

## Conclusions

We investigated whether mutational analysis of samples along the Müllerian tract could be used to detect ovarian cancer. Diagnostic accuracy with our analysis was low for cervicovaginal self-samples, Pap smears, and endometrial biopsies when comparing the pathogenic variants in the samples to the variants in the tumor itself. Thus, these samples should not be used for (early) ovarian cancer detection.

## Data Availability

Data are available upon reasonable request. All data relevant to the study are included in the article or uploaded as supplementary information. The data supporting the conclusions of this article are available upon reasonable request to the corresponding author. In accordance with the journal’s guidelines, we will provide our data for independent analysis by a selected team by the Editorial Team for the purposes of additional data analysis or for the reproducibility of this study in other centers if such is requested.

## References

[1] Sung H , Ferlay J , Siegel RL , et al . Global cancer statistics 2020: GLOBOCAN estimates of incidence and mortality worldwide for 36 cancers in 185 countries. CA Cancer J Clin 2021;71:209–49. 10.3322/caac.21660 33538338

[2] Siegel RL , Miller KD , Jemal A . Cancer statistics, 2019. CA Cancer J Clin 2019;69:7–34. 10.3322/caac.21551 30620402

[3] Menon U , Gentry-Maharaj A , Burnell M , et al . Ovarian cancer population screening and mortality after long-term follow-up in the UK Collaborative Trial of Ovarian Cancer Screening (UKCTOCS): a randomised controlled trial. Lancet 2021;397:2182–93. 10.1016/S0140-6736(21)00731-5 33991479 PMC8192829

[4] Labidi-Galy SI , Papp E , Hallberg D , et al . High grade serous ovarian carcinomas originate in the fallopian tube. Nat Commun 2017;8:1093. 10.1038/s41467-017-00962-1 29061967 PMC5653668

[5] Hermsen BBJ , Olivier RI , Verheijen RHM , et al . No efficacy of annual gynaecological screening in BRCA1/2 mutation carriers; an observational follow-up study. Br J Cancer 2007;96:1335–42. 10.1038/sj.bjc.6603725 17426707 PMC2360170

[6] Kinde I , Bettegowda C , Wang Y , et al . Evaluation of DNA from the Papanicolaou test to detect ovarian and endometrial cancers. Sci Transl Med 2013;5:ra4. 10.1126/scitranslmed.3004952 PMC3757513 23303603

[7] Wang Y , Li L , Douville C , et al . Evaluation of liquid from the Papanicolaou test and other liquid biopsies for the detection of endometrial and ovarian cancers. Sci Transl Med 2018;10:eaap8793. 10.1126/scitranslmed.aap8793 29563323 PMC6320220

[8] Maritschnegg E , Wang Y , Pecha N , et al . Lavage of the uterine cavity for molecular detection of Müllerian duct carcinomas: a proof-of-concept study. J Clin Oncol 2015;33:4293–300. 10.1200/JCO.2015.61.3083 26552420 PMC4678180

[9] Reijnen C , van der Putten LJM , Bulten J , et al . Mutational analysis of cervical cytology improves diagnosis of endometrial cancer: a prospective multicentre cohort study. Int J Cancer 2020;146:2628–35. 10.1002/ijc.32686 31523803

[10] Eijkelenboom A , Kamping EJ , Kastner-van Raaij AW , et al . Reliable next-generation sequencing of formalin-fixed, paraffin-embedded tissue using single molecule tags. J Mol Diagn 2016;18:851–63. 10.1016/j.jmoldx.2016.06.010 27637301

[11] Cancer Genome Atlas Research Network, Kandoth C , Schultz N , et al . Integrated genomic characterization of endometrial carcinoma. Nature 2013;497:67–73. 10.1038/nature12113 23636398 PMC3704730

[12] Cancer Genome Atlas Research Network . Integrated genomic analyses of ovarian carcinoma. Nature 2011;474:609–15. 10.1038/nature10166 21720365 PMC3163504

[13] cfCG . Ovarian Epithelial Tumor - Serous Ovarian Cancer, 2014. Available: http://www.cbioportal.org/

[14] Ghezelayagh TS , Pennington KP , Norquist BM , et al . Characterizing TP53 mutations in ovarian carcinomas with and without concurrent BRCA1 or BRCA2 mutations. Gynecol Oncol 2021;160:786–92. 10.1016/j.ygyno.2020.12.007 33375991 PMC8491988

[15] Massuger L , Roelofsen T , van Ham M . The origin of serous ovarian cancer may be found in the uterus: a novel hypothesis. Med Hypotheses 2010;74:859–61. 10.1016/j.mehy.2009.11.029 20022435

[16] Roelofsen T , van Kempen LCLT , van der Laak JAWM , et al . Concurrent endometrial intraepithelial carcinoma (EIC) and serous ovarian cancer: can EIC be seen as the precursor lesion? Int J Gynecol Cancer 2012;22:457–64. 10.1097/IGC.0b013e3182434a81 22249577

[17] Filho AL , Gonçalves AEP , Martinho O , et al . Liquid-based cytology in DNA-based molecular research: viability and potential application. Anal Quant Cytol Histol 2009;31:395–400. 20698355

[18] Barrett JE , Jones A , Evans I , et al . The DNA methylome of cervical cells can predict the presence of ovarian cancer. Nat Commun 2022;13:448. 10.1038/s41467-021-26615-y 35105887 PMC8807742

[19] Havrilesky LJ , Moorman PG , Lowery WJ , et al . Oral contraceptive pills as primary prevention for ovarian cancer: a systematic review and meta-analysis. Obstet Gynecol 2013;122:139–47. 10.1097/AOG.0b013e318291c235 23743450

[20] Buchta V . Vaginal microbiome. Ceska Gynekol 2018;83:371–9. 30848142

[21] Chambers LM , Bussies P , Vargas R , et al . The microbiome and gynecologic cancer: current evidence and future opportunities. Curr Oncol Rep 2021;23:92. 10.1007/s11912-021-01079-x 34125319

[22] Nené NR , Reisel D , Leimbach A , et al . Association between the cervicovaginal microbiome, BRCA1 mutation status, and risk of ovarian cancer: a case-control study. Lancet Oncol 2019;20:1171–82. 10.1016/S1470-2045(19)30340-7 31300207

[23] Abu-Ghazaleh N , Chua WJ , Gopalan V . Intestinal microbiota and its association with colon cancer and red/processed meat consumption. J Gastroenterol Hepatol 2021;36:75–88. 10.1111/jgh.15042 32198788

[24] Erickson BK , Kinde I , Dobbin ZC , et al . Detection of somatic TP53 mutations in tampons of patients with high-grade serous ovarian cancer. Obstet Gynecol 2014;124:881–5. 10.1097/AOG.0000000000000484 25437714 PMC4316672

[25] Vos JR , Fakkert IE , de Hullu JA , et al . Universal tumor DNA BRCA1/2 testing of ovarian cancer: prescreening PARPi treatment and genetic predisposition. J Natl Cancer Inst 2020;112:161–9. 10.1093/jnci/djz080 31076742 PMC7019087

[26] Maritschnegg E , Heitz F , Pecha N , et al . Uterine and tubal lavage for earlier cancer detection using an innovative catheter: a feasibility and safety study. Int J Gynecol Cancer 2018;28:1692–8. 10.1097/IGC.0000000000001361 30376484 PMC6254778

[27] Moore L , Leongamornlert D , Coorens THH , et al . The mutational landscape of normal human endometrial epithelium. Nature 2020;580:640–6. 10.1038/s41586-020-2214-z 32350471

