# Supplementary material for: Diagnostic accuracy of mutational analysis along the Müllerian tract to detect ovarian cancer
Source: Int J Gynecol Cancer. 2022 Nov 16;32(12):1568–75. doi: 10.1136/ijgc-2022-003911 (PMC9763223; doi:10.1136/ijgc-2022-003911)

Supplementary

Supplementary Document 1. Workflow for analyzing pathogenic variants p. 2

Supplementary Table 1. Gene regions targeted in our single molecule molecular inversion probes panel p. 5

Supplementary Figure 1. Mean numbers of unique reads for each variant region p. 6

Supplementary Table 2. Coverage details per sequenced exon p. 8

Supplementary Table 3. Baseline characteristics of each patient p. 11

Supplementary Table 4. Identified pathogenic variants in the four specimens per patient p. 14

Supplementary Document 2. Detection rate per sampling method by stage p. 17

van Bommel MH.D, *et al. Int J Gynecol Cancer* 2022; 32:1568–1575. doi: 10.1136/ijgc-2022-003911

Supplementary Document 1. *Workflow for analyzing pathogenic variants*

Tissue processing

The cervicovaginal self-sample (Evalyn Brush, Rovers Medical Devices B.V., Oss, the Netherlands) was taken according to the manufacturer's instructions. The brush tips of the cervicovaginal self-sample and Pap smear were suspended in Preservcyt medium (Hologic, Marlborough, MA). The brush tips of the cervicovaginal self-sample were suspended in 4.5ml vials, which were vortexed for 3 x 15s, stored overnight at 4 ^{o}C, and again vortexed for 2 x 15s, before the brushes were removed and discarded. The samples were stored at room temperature until DNA extraction. A representative Formalin-Fixed Paraffin-Embedded surgical sample of the ovary was selected at the end of the surgery in both ovarian cancer patients and in women undergoing surgery for a benign gynecological condition (control group).

DNA Extraction

Representative areas of the ovarian cancer in the surgical specimens were marked and selected by macrodissection from 2 x 20 µm thick FFPE sections (C.R., J.B.). From the pipelle endometrial biopsies, two 20 µm thick sections were fully used for DNA extraction, without selecting tissue by macrodissection. The cervicovaginal self-samples and Pap smears were centrifuged for 5 minutes at 14.000 x g, after which the supernatant was transferred and the pellets were centrifuged for 1 minute at 14.000 x g. The remaining pellet was used for DNA extraction. These specimens were digested at 56°C overnight in TET-lysisbuffer (10mmol/L Tris/HCL pH8.5, 1mmol/L EDTA pH8.0, 0.01% Tween-20) with 5% Chelex-100 (Bio-Rad, Hercules, CA, US) and 0.2% proteinase K, followed by inactivation at 95°C for ten minutes. Subsequently, the supernatant was transferred after centrifugation into a clean tube. DNA concentration was determined using the Qubit Broad Range Kit (Thermo Fisher Scientific, Waltham, MA, US).

Single molecule molecular inversion probes panel design and library preparation

The samples were analyzed using single molecule Molecular Inversion Probes (Integrated DNA Technologies, Leuven, Belgium). The design of the single molecule Molecular Inversion Probes and the library preparation were performed as previously described [10]. Briefly, single molecule Molecular Inversion Probes were designed in a tiling manner for all included hotspots in oncogenes relevant in endometrial and ovarian cancer (*CTNNB1, KRAS, MTOR, PIK3CA* and *POLE*) and all coding and splice site sequences of tumor suppressor genes (*ARID1A, PTEN,* and *TP53*, **Supplementary Table**

1) preferentially targeting both strands with two independent single molecule Molecular Inversion Probes (probe sequences available on request). The single molecule Molecular Inversion Probes consisted of an extension and ligation probe arm, together 40bp long, with a gap of 112 bp, with a common backbone sequence for PCR-based library amplification. The ligation probe arm and backbone are connected with a backbone, also containing an 8bp degenerate sequence (8xN) serving as a Unique Molecular Identifier (UMI, also known as 'single molecule tag'). The single molecule Molecular Inversion Probes were mixed and phosporylated using 1 µL of T4 polynucleotide kinase (M0201; New England Biolabs, Ipswich, MA, US) per 25 µL of 100 µmol/L smMIPs and ATP-containing G4 DNA ligase buffer (B0202, New England Biolabs). The molecular ratio between gDNA and single molecule Molecular Inversion Probes was 1:3200 for every individual smMIP and the standard genomic DNA input was set at 100 ng.

A capture mix was made (total capture volume 25 µL) containing the phosporylated smMIP pool, 1 unit of Ampligase DNA ligase (A0110K; EpiBio, Madison, WI) and Ampligase Buffer (A1905B, DNA ligase buffer), 3.2 units of Hemo Klentaq (M0332; New England Biolabs), finally 8 mmol of dNTPs (28-4065-20/-12/-22/-32; GE Healthcare, Little Chalfont, UK) and, when available, 100 ng of genomic DNA in a 20 µL volume. Subsequently, this capture mix was denatured (95°C for 10 minutes) and incubated for probe hybridization, extension and ligation at 60°C for 18 hours. After cooling, exonuclease treatment was performed by adding Exonuclease I (10 units; M0293; New England Biolabs) and III (50 units; M0206; New England Biolabs) and Ampligase Buffer to the capture mix (total of 27 µL) and incubating at 37°C for 45 minutes, with subsequent inactivation at 95°C for 2 minutes. A total of 20 µL was used for PCR in a total volume of 50 µL including a common forward primer, bar-coded reverse primers, and iProof high fidelity master mix (1725310, Bio-Rad, Veenendaal, the Netherlands). The resulting PCR products were pooled prior to purification with 0.8x volume of Agencourt Ampure XP Beads (A63881, Beckman Coulter, Woerden, the Netherlands). Each cytology sample was assessed in two independent library preparations, because of the expected low mutant allele frequencies.

Sequencing and analysis

Sequencing of the purified libraries, denatured and diluted to 1.2pmol/L, was performed on a NexSeq500 device (Illumina, San Diego, CA, US) according to the manufacturer's instructions (300 cycles High Output sequencing kit, v2), resulting in 2x150bp paired-end reads. The resulting Bcl files were converted to fastq files and bar-coded reads were subsequently demultiplexed. Single-molecule-directed assembly of duplicate reads was performed to generate consensus ('unique') reads using the analysis software Sequence Pilot (version 4.4.0; JSI medical systems, Ettenheim,

Germany). For variant calling in Sequence Pilot, variant detection thresholds were generally set to 3% (surgical specimens and pipelles) or 1% (Pap smears and self-samples) of all unique reads at that position and a minimum of 5 unique reads representing ≥3 individual gDNA molecules.

Variants were classified as “pathogenic”, “likely pathogenic”, “variant of unknown significance”, “likely benign” and “benign” and the first three categories were considered (potentially) pathogenic. Synonymous variants were only considered when present at exon ends. Finally, intronic variants were excluded with the exception of splice site sequences. First, the surgical specimens (of ovarian cancer or a benign gynecological condition) were analyzed for the presence of somatic variants using variant calling. Subsequently, these variants were investigated in pipelle endometrial biopsies and the cytology samples, using a three-step method. First, variants yielded by variant calling were assessed. Second, all samples were manually screened for the presence of reads harboring the known variants. In case no variant was found in the surgical specimen, the cytology samples were searched for the presence of known hotspot variants in *CTNNB1, KRAS, MTOR, PIK3CA* and *POLE*.

After this, assessment of variants in the pipelle endometrial biopsies and cytology samples was performed, with adjusted variant calling settings for hotspots in oncogenes (minimum of 5 unique reads representing 3 gDNA molecules, without minimal variant allele frequencies). Settings for tumor suppressor genes were unadjusted.

To determine whether sufficient DNA molecules were sequenced to reliably (>95% certainty) exclude variants above a certain mutant allele frequency, a cumulative binomial distribution was used that calculated the required unique read depths [10]. For all surgical specimens and pipelle endometrial biopsies these required read depths were assessed in the context of the estimated tumor load (percentage of neoplastic cells). For all cytology samples, a unique read depth representing > 250 individual gDNA molecules at each hotspot position was pursued, in order to reduce the chance to less than 5% of missing variants with an variant allele frequency above 3%. In case hotspot positions were sequenced with insufficient unique read depth, library preparation and sequencing was repeated (once or twice if needed). Mean numbers of unique reads for each variant region are shown in **Supplementary Figure 1**.

In order to exclude potential false positive calls due to PCR, sequencing, or Formalin-Fixed Paraffin-Embedded deamination artifacts, the background was determined at all sequenced positions using 12 control Pap smears and cervicovaginal self-samples. Because proliferating endometrium could harbor PIK3CA and KRAS mutations, only cytology samples from patients with histo-pathologically proven atrophic endometrium were selected. The variant allele frequency to background (signal to noise) ratio was > 5 for all identified variants.

van Bommel MH.D, *et al. Int J Gynecol Cancer* 2022; 32:1568–1575. doi: 10.1136/ijgc-2022-003911

Supplementary Table 2. *Gene regions targeted in our single molecule Molecular Inversion Probes panel*

**Gene**	**Exon**	**Targeted codons**	**Positions**	**RefSeq ID**	**Ensembl ID**
ARID1A	1 to 20	M1-Stop2286	c.1 to c.6858	NM_006015.5	ENST00000324856
CTNNB1	3	D32-S45	c.53 to c.146	NM_001904.3	ENST00000349496
KRAS	2	G12-G13	c.9 to c.71	NM_004985.4	ENST00000311936
	3	A59-Q61	c.122 to c.215		
	4	K117, A146	c.291-5 to c.357		
			c.402 to c.450+5		
MTOR	30	D1458-E1489	c.4371 to c.4469+5	NM_004958.3	ENST00000361445
	39	A1789-A1820	c.5365-5 to c.5460		
	43	A1971-L1995	c.5911-5 to c.5985		
	47	Q2194-L2220	c.6580 to c.6662+5		
	53	M2404-D243	c.7210 to c.7300+5		
	56	G2484-T2509	c.7448-5 to c.7527		
PIK3CA	10	E542-Q546	c.1558 to c.1664+5	NM_006218.3	ENST00000263967
	21	M1043-G1049	c.3058 to c.3207+10		
POLE	9 to 14	D268-E491	c.802-5 to c.1473+5	NM_006231.3	ENST00000320574
PTEN	1 to 9	M1-Stop404	c.1 to c.1210+5	NM_000314.6	ENST00000371953
TP53	2 to 11	>95% of all coding and splice sequences (-5/+5)	c.1 to c.1180+5	NM_000565.5	ENST00000269305

van Bommel MH.D, *et al. Int J Gynecol Cancer* 2022; 32:1568–1575. doi: 10.1136/ijgc-2022-003911

Supplementary Figure 1. *Mean numbers of unique reads for each variant region*

A. Ovarian cancer patients (n=29)

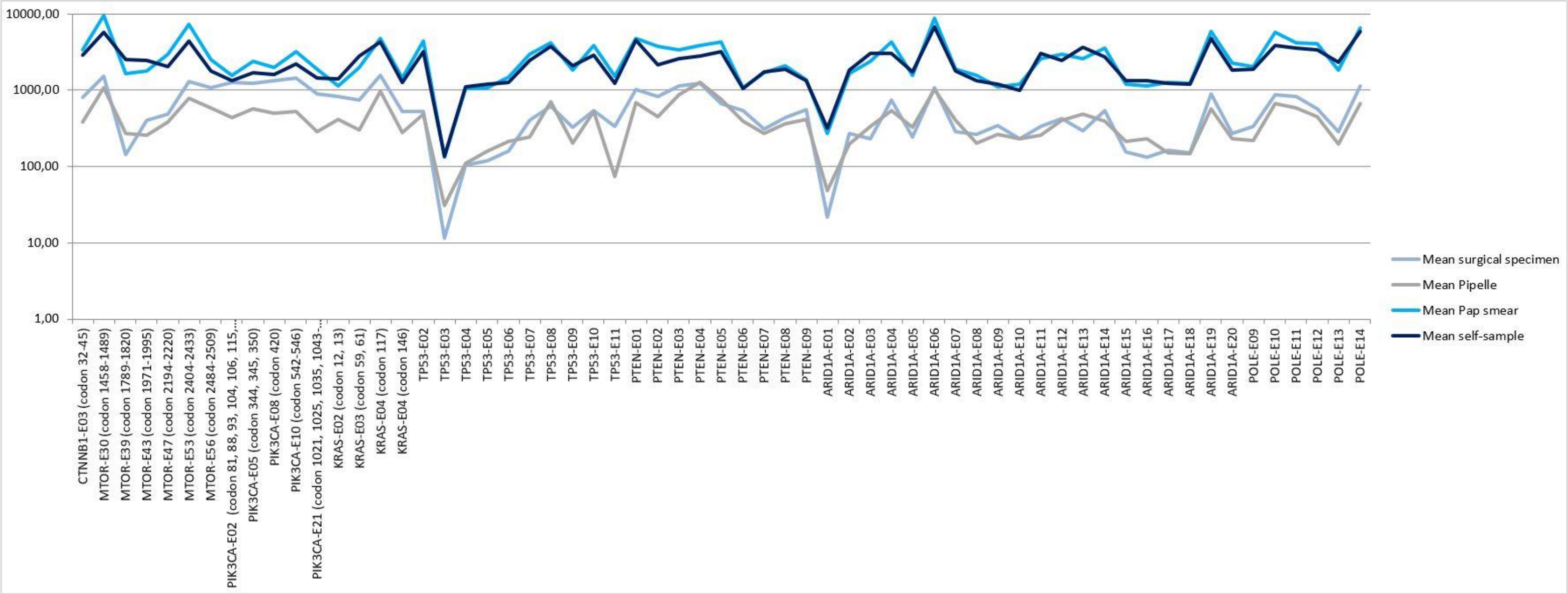

B. Control patients (n=32)

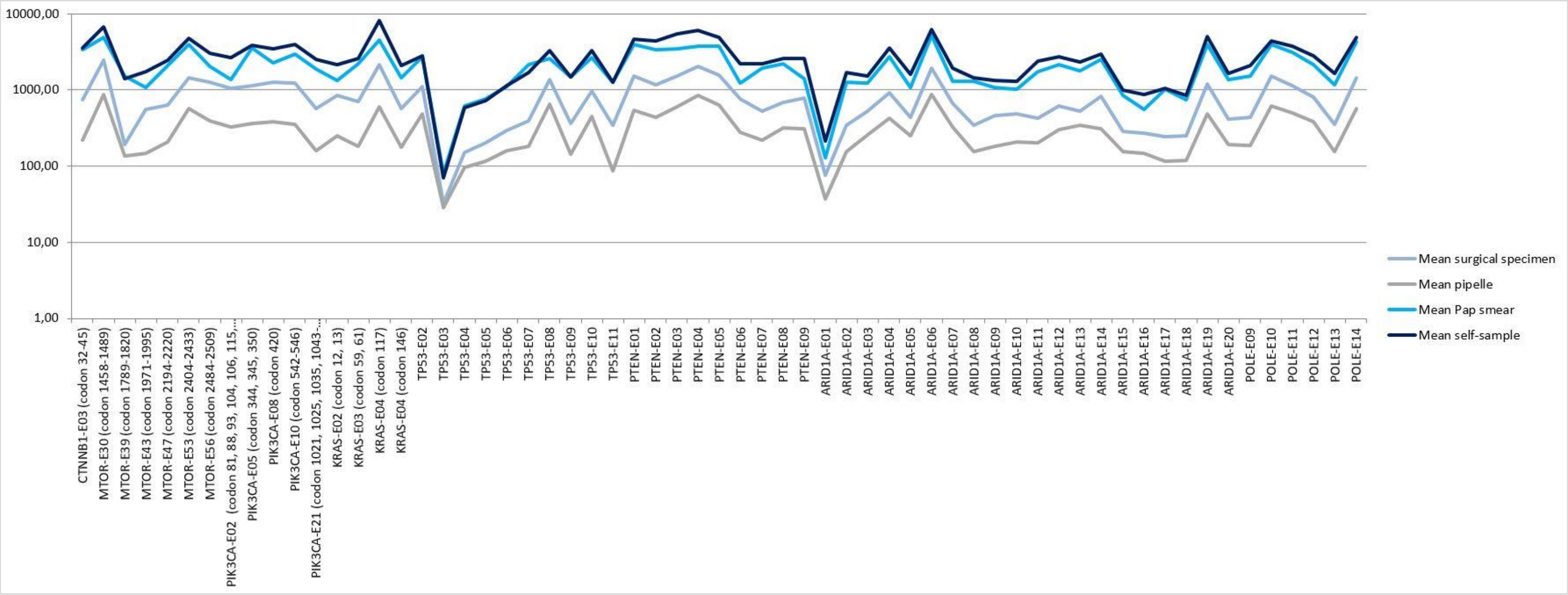

Supplementary Table 2. Coverage details per sequenced exon

Exon	Ovarian cancer patients												Control patients											
	Self-sample			Pap smear			Pipelle			Surgical specimen			Self-sample			Pap smear			Pipelle			Surgical specimen		
	Mean	P25	P75	Mean	P25	P75	Mean	P25	P75	Mean	P25	P75	Mean	P25	P75	Mean	P25	P75	Mean	P25	P75	Mean	P25	P75
CTNNB1-E03 (codon 32-45)	2861.5	872.5	4443.5	3408.4	1854.0	4575.0	387.0	35.0	562.5	796.6	122.5	1139.3	3597.7	1755.3	4332.3	3381.1	1578.0	4083.0	222.2	36.0	346.0	753.4	408.0	786.0
MTOR-E30 (codon 1458-1489)	5759.2	1361.0	7908.0	9585.1	2979.0	15141.0	1068.9	96.0	1523.0	1536.1	420.0	1979.8	6746.8	2850.0	7926.5	4944.3	2660.0	6212.0	866.0	142.0	1322.0	2497.9	1486.0	2946.0
MTOR-E39 (codon 1789-1820)	2510.0	416.0	3382.0	1658.7	800.0	2288.0	274.9	13.0	412.0	145.4	24.5	210.0	1416.7	611.5	2032.3	1518.9	557.0	1908.0	137.7	16.0	186.0	191.8	72.0	180.0
MTOR-E43 (codon 1971-1995)	2476.8	1136.5	3708.0	1805.4	865.0	2678.0	255.1	17.0	364.0	400.2	83.8	573.3	1727.1	988.0	2028.5	1081.7	556.0	1610.0	146.5	20.0	214.0	551.0	352.0	682.0
MTOR-E47 (codon 2194-2220)	2037.1	722.5	3208.0	2955.1	1344.0	3986.0	380.1	24.0	537.0	491.4	107.5	609.0	2488.6	1344.5	2917.5	2116.6	1239.0	2591.0	208.1	56.0	308.0	640.9	368.0	689.0
MTOR-E53 (codon 2404-2433)	4373.2	998.0	5537.5	7281.9	1604.0	12030.0	786.3	64.0	1114.0	1294.8	316.0	1494.5	4831.7	1634.5	6646.0	3940.9	2118.0	5273.0	576.1	148.0	838.0	1428.6	972.0	1950.0
MTOR-E56 (codon 2484-2509)	1801.1	434.0	2466.0	2542.4	675.0	3796.0	585.1	47.0	754.0	1084.8	202.0	1339.0	3067.8	1312.5	4230.8	2043.2	620.0	3022.0	393.8	40.0	660.0	1272.3	762.0	1724.0
PIK3CA-E02 (codon 81, 88, 93, 104, 106, 115, 118)	1326.9	378.0	1886.0	1574.0	300.0	1930.0	435.3	24.0	620.0	1278.4	278.0	1355.0	2637.3	1007.0	3315.0	1353.8	480.0	1906.0	331.0	32.0	558.0	1059.9	684.0	1396.0
PIK3CA-E05 (codon 344, 345, 350)	1715.9	895.5	2369.0	2428.5	862.0	3204.0	571.1	44.0	776.0	1247.0	372.0	1217.8	3854.3	2400.0	5360.3	3527.1	1926.0	5059.0	368.0	54.0	580.0	1130.5	714.0	1284.0
PIK3CA-E08 (codon 420)	1611.9	400.5	2536.5	2002.8	570.0	2554.0	495.0	38.0	692.0	1342.9	362.0	1417.5	3455.3	1652.8	4912.3	2291.7	796.0	3704.0	383.7	28.0	652.0	1254.2	790.0	1674.0
PIK3CA-E10 (codon 542-546)	2219.6	705.0	3184.0	3245.4	1293.0	4495.0	523.5	47.0	739.0	1445.8	339.8	1409.0	3963.1	1901.0	5747.0	2974.8	1424.0	3986.0	352.2	44.0	590.0	1235.1	741.0	1583.0
PIK3CA-E21 (codon 1021, 1025, 1035, 1043-1049, 1069)	1465.3	564.0	2378.5	1906.0	1072.0	2696.0	290.1	30.0	420.0	904.0	190.5	965.0	2513.1	1532.3	3545.8	1897.0	1037.0	2356.0	161.1	18.0	260.0	567.4	340.0	680.0
KRAS-E02 (codon 12, 13)	1398.0	301.0	1808.0	1147.6	130.0	1722.0	418.7	29.0	586.0	822.1	151.0	1072.0	2147.2	833.0	3126.5	1322.5	312.0	2101.0	252.6	18.0	418.0	860.4	554.0	1182.0
KRAS-E03 (codon 59, 61)	2804.1	1264.5	4135.5	1996.5	1118.0	2744.0	304.0	38.0	449.0	746.0	107.5	1095.0	2599.2	1447.8	3718.3	2218.3	1004.0	3010.0	184.3	18.0	264.0	715.0	420.0	858.0
KRAS-E04 (codon 117)	4252.0	1758.5	6797.0	4795.2	2433.0	5970.0	971.7	77.0	1248.0	1557.4	438.0	2006.5	8186.1	4196.5	11352.5	4554.1	2827.0	6478.0	609.5	64.0	952.0	2163.5	1376.0	2549.0
KRAS-E04 (codon 146)	1274.2	367.0	1889.5	1441.8	398.0	2242.0	282.2	19.0	399.0	523.6	133.5	645.5	2123.8	946.8	3088.5	1456.4	446.0	2176.0	176.1	16.0	290.0	577.9	370.0	757.0
TP53-E02	3190.1	1246.7	5058.8	4406.8	3314.6	5814.9	490.6	59.8	850.7	531.1	128.6	532.4	2802.1	1283.6	4023.1	2705.8	1475.4	3187.6	491.5	114.0	591.0	1105.1	550.0	1370.5
TP53-E03	136.9	60.0	155.0	133.7	56.0	202.0	31.2	2.0	46.0	11.6	2.0	14.0	70.5	35.5	101.5	79.6	58.0	94.0	28.6	6.0	46.0	32.6	14.0	46.0
TP53-E04	1101.2	514.9	1679.7	1056.9	699.1	1469.5	111.4	15.0	194.9	105.1	29.0	128.8	584.0	291.1	827.7	623.0	346.9	830.4	96.5	21.7	126.2	153.1	99.0	205.0
TP53-E05	1212.3	363.7	1658.7	1080.1	353.3	1626.2	159.6	22.4	257.5	118.0	25.8	157.5	732.8	347.2	1014.2	764.0	348.1	1064.6	114.8	37.3	154.8	201.3	96.6	205.1

TP53-E06	1282.0	543.9	1960.0	1498.4	879.0	2173.8	214.8	28.8	373.6	157.9	33.9	173.0	1143.6	586.2	1475.0	1123.5	597.4	1456.4	159.5	35.3	223.6	294.7	161.5	284.6
TP53-E07	2464.8	1134.9	3813.8	2977.0	1745.9	3786.5	243.9	27.5	387.2	405.2	94.0	369.0	1673.7	741.8	2148.1	2163.2	756.2	2815.6	180.7	43.2	247.2	391.8	187.3	503.8
TP53-E08	3752.0	1730.3	5399.1	4196.0	2099.1	5729.4	708.0	73.5	1208.4	615.0	145.6	766.7	3267.1	1934.8	4527.0	2577.8	1624.3	3195.9	648.3	174.7	862.9	1377.4	844.4	1617.8
TP53-E09	2081.6	883.4	2867.1	1837.4	1077.7	2666.9	200.8	18.8	336.2	326.3	75.6	397.7	1489.6	834.8	2025.0	1472.2	765.4	2088.4	143.0	31.0	181.6	366.5	230.9	457.7
TP53-E10	2889.5	784.6	4786.0	3884.5	2196.3	4888.3	529.6	46.5	875.9	542.0	141.7	707.6	3295.2	1697.9	4373.0	2645.0	1515.9	3412.9	454.9	111.1	547.8	971.5	539.6	1050.4
TP53-E11	1233.2	622.6	1493.9	1508.4	972.7	2215.9	73.8	12.3	106.9	334.9	103.0	462.8	1264.2	757.8	1641.1	1266.9	633.3	1451.0	87.1	15.4	83.2	343.1	155.9	388.9
PTEN-E01	4572.0	2469.6	6255.9	4722.7	3209.1	6046.1	693.4	86.7	1149.8	1019.7	249.2	1286.0	4715.7	3053.0	5921.6	3966.5	2577.1	5144.4	545.6	111.3	652.4	1530.2	964.9	1681.8
PTEN-E02	2129.7	1161.6	2947.7	3737.4	2058.7	4881.2	453.3	37.8	645.8	835.0	205.2	1047.9	4465.2	2460.5	6160.4	3353.6	1609.8	4641.8	438.6	60.9	569.7	1155.6	661.7	1289.4
PTEN-E03	2583.2	788.0	3736.5	3419.2	747.0	5184.0	871.7	46.0	1196.0	1130.8	334.0	1641.8	5496.7	2404.5	7613.8	3480.0	935.0	5685.0	601.9	64.0	826.0	1505.5	916.0	1882.0
PTEN-E04	2811.1	905.5	3619.0	3859.6	894.0	5950.0	1254.7	80.0	1943.0	1250.0	399.5	1732.5	6036.4	2813.0	9348.8	3774.7	1022.0	6208.0	843.5	126.0	1164.0	2053.9	1020.0	2343.0
PTEN-E05	3174.1	1502.1	5304.4	4253.1	2354.9	5383.2	768.4	61.8	1167.2	667.5	234.7	755.6	4844.3	2597.3	6325.7	3766.8	2176.6	4769.4	635.1	124.9	836.7	1584.5	916.0	1602.4
PTEN-E06	1049.5	428.0	1390.1	1075.7	350.4	1429.2	389.2	25.8	573.5	537.1	137.0	666.9	2223.6	1302.4	2884.1	1230.7	460.8	1747.7	278.4	21.8	411.1	765.8	313.9	905.9
PTEN-E07	1741.5	836.3	2614.2	1707.5	1173.7	2054.1	273.2	32.9	390.3	310.9	105.6	404.1	2208.3	1215.4	2945.4	1937.8	1129.0	2130.9	217.6	27.2	253.9	520.1	308.5	590.4
PTEN-E08	1864.1	946.7	2601.4	2121.2	1367.5	2787.4	367.8	38.1	534.0	438.2	162.7	558.3	2589.3	1542.5	3434.7	2188.7	1326.5	2780.6	313.9	50.0	440.2	691.8	425.0	853.3
PTEN-E09	1341.7	433.5	1822.7	1372.7	428.6	2171.5	413.0	30.1	546.6	557.9	180.8	736.1	2610.7	1329.3	3459.5	1423.6	773.7	1963.3	305.8	41.1	399.3	779.3	477.1	888.8
ARID1A-E01	318.8	113.9	420.9	271.6	126.9	400.6	48.9	5.8	61.8	21.9	7.2	36.5	214.2	109.5	253.8	129.8	62.6	162.0	37.3	12.9	50.8	75.8	38.8	86.1
ARID1A-E02	1838.2	600.1	2835.2	1671.7	1203.1	2146.9	199.7	18.5	301.2	271.7	73.9	362.6	1694.0	1034.1	2050.4	1273.5	703.7	1595.6	154.8	37.2	175.0	342.0	220.2	390.8
ARID1A-E03	3052.3	1451.8	4105.8	2424.9	1312.6	3569.5	334.8	44.2	569.7	234.5	58.5	261.1	1524.3	798.5	2124.7	1240.4	742.5	1725.2	254.0	71.7	336.0	524.0	297.5	537.6
ARID1A-E04	3021.1	1131.3	4525.8	4336.7	2172.9	6045.6	547.0	55.6	812.1	751.7	168.7	952.5	3574.4	1968.8	4664.6	2772.1	1666.7	3833.6	430.7	72.3	479.0	917.6	585.2	1114.9
ARID1A-E05	1759.3	669.4	2763.3	1570.6	994.9	2118.5	328.5	30.1	529.9	245.7	57.8	304.2	1597.9	959.3	2160.3	1093.7	609.8	1518.9	250.3	58.7	305.7	437.6	255.7	481.5
ARID1A-E06	6811.6	2524.2	10819.3	8718.4	4741.8	13472.0	1036.0	107.2	1634.0	1070.2	269.3	1372.0	6204.8	3765.6	7308.9	5193.8	2945.4	6536.8	873.6	238.0	1105.8	1927.9	1082.9	2295.2
ARID1A-E07	1785.2	702.0	2439.4	1884.9	950.9	2439.8	408.9	39.3	671.6	286.1	85.7	398.2	1917.1	1165.7	2673.5	1296.0	722.2	1670.5	323.9	81.7	440.5	672.6	448.1	718.2
ARID1A-E08	1346.9	483.3	2144.2	1582.8	1187.7	2043.8	203.9	20.7	315.3	264.3	68.8	334.7	1449.5	813.5	1829.3	1302.6	676.1	1694.2	155.5	30.1	194.0	346.0	201.1	393.9
ARID1A-E09	1209.3	500.2	1556.2	1121.0	708.2	1540.9	262.6	24.9	406.3	342.4	81.9	478.6	1340.9	713.1	1953.5	1085.4	655.3	1402.6	183.0	25.4	194.9	455.4	280.7	550.2
ARID1A-E10	1007.5	598.5	1256.7	1214.9	774.2	1549.8	230.2	25.3	386.1	230.7	59.5	285.2	1301.1	808.1	1799.1	1022.5	701.9	1375.6	207.8	44.0	296.2	491.6	283.8	589.3
ARID1A-E11	3023.7	1074.7	4673.5	2614.6	1583.1	3535.4	257.0	28.0	366.6	333.3	78.0	442.4	2426.7	1379.7	3051.8	1737.4	940.8	2174.3	200.9	45.2	230.9	430.4	242.6	474.8
ARID1A-E12	2464.6	1164.9	4001.5	2979.6	1939.6	4023.9	406.8	44.1	642.1	429.8	135.5	535.1	2720.7	1472.8	3539.4	2132.9	1322.8	2755.1	305.6	57.4	356.1	623.0	370.8	687.7
ARID1A-E13	3641.2	1567.0	5298.0	2574.0	1106.5	3911.0	487.2	58.6	820.3	291.8	73.5	417.6	2330.4	1134.6	3054.7	1775.4	1032.7	2309.9	341.3	80.8	440.2	533.5	249.8	636.1
ARID1A-E14	2765.4	1231.4	4329.0	3550.6	2651.0	4769.6	396.7	52.3	599.0	541.8	119.3	672.4	2951.6	1662.2	3895.9	2554.1	1544.5	3357.3	313.7	73.1	390.3	824.1	476.5	785.4
ARID1A-E15	1352.7	453.0	2121.9	1197.1	632.9	1617.2	211.6	21.6	355.3	154.0	41.6	216.2	1001.7	449.4	1294.1	838.9	410.2	1162.9	153.3	28.6	214.7	288.2	166.0	273.7

ARID1A-E16	1324.4	608.6	1957.8	1155.0	728.7	1899.8	232.8	22.7	402.3	134.0	31.8	195.1	871.6	473.4	1162.0	561.3	328.2	770.3	147.9	41.4	220.2	274.1	161.5	289.9
ARID1A-E17	1222.8	426.0	2017.8	1259.3	635.8	1739.5	153.3	16.6	240.5	165.5	34.4	221.3	1039.8	480.2	1568.9	1011.6	450.0	1272.7	116.7	27.9	160.1	243.5	131.4	247.6
ARID1A-E18	1203.3	440.1	1812.9	1221.1	722.2	1549.9	147.7	19.1	250.7	153.3	39.7	184.4	841.7	505.3	1075.0	739.7	512.4	923.5	120.1	28.4	157.1	247.6	147.1	272.8
ARID1A-E19	4809.4	1504.9	7174.3	5997.1	2456.5	8553.2	574.8	57.4	860.7	901.3	181.2	1035.9	4990.0	2897.5	6147.3	3921.6	2066.7	5136.4	483.6	82.5	541.2	1215.3	654.2	1141.3
ARID1A-E20	1859.0	675.1	3026.4	2257.9	1677.6	3086.6	229.9	30.2	360.1	272.3	70.3	320.8	1669.3	909.5	1986.1	1387.5	829.4	1789.9	193.3	42.8	232.8	418.1	258.8	419.8
POLE-E09	1873.4	818.8	2893.4	2036.2	1559.1	2915.9	221.8	18.4	355.6	338.8	97.8	415.9	2098.9	1296.7	2729.6	1525.2	845.6	1913.0	184.6	30.6	208.8	435.7	239.0	514.7
POLE-E10	3853.3	1564.1	5809.0	5764.9	2816.0	8392.7	673.8	62.7	1104.9	874.2	230.6	1374.3	4446.4	2563.0	5797.0	4015.1	2128.0	4787.3	612.7	133.7	768.9	1508.8	864.8	1726.7
POLE-E11	3543.9	1289.3	5650.8	4197.8	2087.1	6443.6	585.9	71.4	895.7	828.0	191.2	1188.2	3808.2	2327.9	4546.5	3127.6	1740.5	4133.1	504.6	103.4	544.9	1125.0	634.1	1431.1
POLE-E12	3399.6	883.6	5971.3	4066.8	2167.3	6869.9	449.9	54.6	725.1	571.2	131.2	834.8	2841.2	1251.4	3479.2	2147.4	1250.0	2706.8	386.2	86.9	451.8	813.1	475.9	975.3
POLE-E13	2362.9	919.8	2892.7	1845.1	1008.5	2892.4	196.7	17.0	316.4	285.5	67.5	361.5	1659.6	950.3	1728.5	1168.3	631.7	1427.4	155.4	42.8	158.6	349.2	220.3	471.4
POLE-E14	5913.6	2696.5	9011.5	6653.9	3967.5	9931.1	675.5	73.6	1070.7	1149.4	196.8	1693.0	4962.1	2952.0	5940.0	4307.1	2299.5	5670.8	567.9	124.1	675.7	1451.6	745.0	1688.5
Total mean	2414.1	918.4	3578.8	2832.6	1412.9	4032.3	422.6	39.5	639.9	600.9	149.0	753.5	2759.8	1450.6	3645.0	2139.8	1107.1	2851.6	321.5	60.1	432.6	814.7	475.8	955.4
Percentage of exons with mean coverage >250 reads	98%			98%			71%			79%			97%			97%			54%			89%		

P25, 25th percentile; P75, 75th percentile.
A mean coverage of >250 reads reflects a probability of 95% to detect a pathogenic variant [1]

[1] Eijkelenboom A, Kamping EJ, Kastner-van Raaij AW, Hendriks-Cornelissen SJ, Neveling K, Kuiper RP, et al. Reliable Next-Generation Sequencing of Formalin-Fixed, Paraffin-Embedded Tissue Using Single Molecule Tags. J Mol Diagn. 2016;18(6):851-63.

***Supplementary Table 3**. Baseline characteristics of each patient*

Patient ID	Age	Histological diagnosis	Stage	Variants in surgical specimen	Variants in self-sample	Overlapping variants: surgical specimen and self-sample	Variants in Pap smear	Overlapping variants: surgical specimen and Pap smear	Variants in pipelle	Overlapping variants: surgical specimen and pipelle	Note
**Ovarian cancer patients**											
8	76	HGSC	3B	2	0	0	0	0	0	0	
10	41	MMMT	3B	1	0	0	0	0	0	0	
18	70	HGSC	3C	1	0	0	1	1	0	0	
20	65	HGSC	2A	1	0	0	1	1	0	0	
32	*49*	*Borderline*	*NA*	*0*	*0*	*0*	*0*	*0*	*0*	*0*	*Excluded**
39	*71*	*Borderline serous*	*NA*	*0*	*0*	*0*	*0*	*0*	*0*	*0*	*Excluded**
40	66	HGSC	3C	0	2	0	1	0	1	0	
44	32	HGSC	3C	0	0	0	1	0	0	0	
45	60	HGSC	2B	1	0	0	*NA*	*NA*	0	0	
49	50	Clear cell	3B	0	0	0	0	0	0	0	
54	*61*	*HGSC*	*4*	*0*	*0*	*0*	*0*	*0*	*0*	*0*	*Excluded**
55	42	HGSC	3C	0	1	0	0	0	1	0	
58	55	Clear cell/serous	3A	0	1	0	1	0	0	0	
63	80	HGSC	1C	1	0	0	*NA*	*NA*	0	0	
65	58	HGSC	3C	1	0	0	0	0	*NA*	*NA*	
72	59	Clear cell/serous	4	1	0	0	0	0	0	0	
77	75	HGSC	3C	1	0	0	0	0	*NA*	*NA*	
85	59	HGSC	3C	1	0	0	0	0	0	0	
86	*67*	*LGSC*	*4*	*0*	*NA*	*NA*	*0*	*0*	*NA*	*NA*	*Excluded**
107	71	HGSC	4	1	0	0	*NA*	*NA*	1	0	
114	*77*	*HGSC*	*99*	*0*	*1*	*0*	*NA*	*NA*	*0*	*0*	*Excluded**
124	66	HGSC	3C	1	0	0	0	0	0	0	
127	54	Clear cell/ endometrioid	1C	2	0	0	3	1	*NA*	*NA*	

van Bommel MH.D, *et al. Int J Gynecol Cancer* 2022; 32:1568–1575. doi: 10.1136/ijgc-2022-003911

130	66	Adenosquamous	2B	2	0	0	0	0	*NA*	*NA*	
135	62	HGSC	3B	2	1	1	0	0	4	1	
151	69	HGSC	3C	1	0	0	2	1	0	0	
160	61	HGSC	3C	1	0	0	0	0	0	0	
164	*58*	*HGSC*	*3C*	*0*	*0*	*0*	*0*	*0*	*NA*	*NA*	*Excluded**
165	74	Endometrioid	4	2	0	0	0	0	1	0	
167	69	Serous/ endometrioid	1A	3	0	0	0	0	1	0	
168	83	Endometrioid	2A	1	1	0	1	1	*NA*	*NA*	
181	*76*	*HGSC*	*4*	*0*	*0*	*0*	*NA*	*NA*	*NA*	*NA*	*Excluded**
184	69	HGSC	3C	1	1	0	1	0	1	0	
188	68	HGSC	3C	1	0	0	0	0	0	0	
196	79	HGSC	3C	2	*NA*	*NA*	0	0	*NA*	*NA*	
197	*66*	*HGSC*	*3C*	*0*	*NA*	*NA*	*0*	*0*	*NA*	*NA*	*Excluded**
198	65	HGSC	3C	3	*NA*	*NA*	3	3	0	0	
**Control patients**											
4	53	Myoma	-	0	0	0	0	0	0	0	
12	59	Mucinous cystadenoma	-	0	0	0	0	0	0	0	
28	57	Prolapse	-	0	0	0	0	0	0	0	
30	66	Simple cystadenoma	-	0	0	0	0	0	0	0	
33	71	Simple cystadenoma	-	0	0	0	0	0	0	0	
34	63	Teratoma	-	0	0	0	0	0	0	0	
38	53	Fibroma	-	0	0	0	1	0	0	0	
53	51	Teratoma	-	0	0	0	0	0	0	0	
60	57	Mucinous cystadenoma	-	0	0	0	0	0	1	0	
62	60	Fibroma	-	0	0	0	0	0	0	0	
67	45	Adenomyosis	-	0	0	0	0	0	0	0	

68	63	Mucinous cystadenoma	-	1	0	0	0	0	0	0	
73	51	Myoma	-	0	0	0	0	0	0	0	
74	72	Mucinous cystadenoma	-	1	0	0	0	0	0	0	
76	59	Myoma	-	0	0	0	0	0	0	0	
89	53	Teratoma	-	0	0	0	0	0	0	0	
102	48	Myoma	-	0	0	0	0	0	0	0	
104	53	Teratoma	-	0	0	0	0	0	0	0	
115	63	Mucinous cystadenoma	-	0	0	0	0	0	0	0	
116	58	Myoma	-	0	0	0	0	0	0	0	
125	51	Mucinous cystadenoma	-	0	0	0	0	0	0	0	
129	72	Serous cystadenoma	-	0	0	0	0	0	0	0	
131	52	Myoma	-	0	0	0	0	0	0	0	
133	62	Normal	-	0	0	0	0	0	0	0	
136	48	Myoma	-	0	0	0	0	0	0	0	
144	50	Myoma	-	0	0	0	0	0	0	0	
150	46	Myoma	-	0	1	0	0	0	1	0	
153	47	Normal	-	0	0	0	1	0	0	0	
159	74	Prolapse	-	0	0	0	0	0	0	0	
166	82	Inflammation	-	0	0	0	0	0	0	0	
183	70	Fibroma	-	0	0	0	0	0	0	0	
195		cystadenoma	-	*NA*	*NA*	*NA*	0	*NA*	0	*NA*	

** Excluded from analysis as sequencing of the surgical specimen was unsuccessful: coverage was too low to potentially detect any variant*
HGSC, high-grade serous cancer; LGSC, low-grade serous cancer; MMMT, Mixed Müllerian Tumor; NA, not applicable

van Bommel MH.D, *et al. Int J Gynecol Cancer* 2022; 32:1568–1575. doi: 10.1136/ijgc-2022-003911

Supplementary Table 4. *Identified pathogenic variants in the four specimens per patient*

**Ovarian cancer patients**						
**Patient ID**	**Pathogenic variants**	**Pathogenic class**	**VAF in self-sample % (mutant reads)**	**VAF in Pap smear % (mutant reads)**	**VAF in pipelle % (mutant reads)**	**VAF in surgical specimen % (mutant reads)**
8	PTEN:1008C>A p.(Tyr336*)	5	no	no	no	5.2 (38)
8	TP53:c.747G>T p.(Arg249Ser)	5	no	no	no	53.0 (184)
10	PTEN:c.389G>A p.(Arg130Gln)	5	no	no	no	5.7 (17)
18	TP53:c.743G>C p.(Arg248Pro)	5	no	0.37 (10)	no	52.0 (79)
20	TP53:c.743G>A p.(Arg248Gln)	4	no	0.46 (14)	no	100.0 (20)
*32**	none		no	no	no	no
*39**	none		no	no	no	no
40	PIK3CA:c.1633G>A p.(Glu545Lys)	5	1.3 (8)	no	no	no
40	TP53:c.*6T>C(3'UTR)		1.0(18)	5.4 (300)	8.8 (14)	no
44	TP53:c.574C>T p.(Gln192*)	5	no	1.4 (36)	no	no
45	TP53:c.814G>A p.(Val272Met)	5	no	NA	no	56.0 (379)
49	none		no	no	no	no
*54**	none		no	no	no	no
55	KRAS:c.37G>T p.(Gly13Cys)	5	no	no	5.7 (97)	no
55	PIK3CA:c.1624G>A p.(Glu542Lys)	5	5.6 (32)	no	no	no
58	PIK3CA:c.1634A>G p.(Glu545Gly)	5	no	1.5 (19)	no	no
58	TP53:c.542G>A p.(Arg181His)	4	3.4 (18)	no	no	no
63	TP53:c.*6T>C(3'UTR)		no	NA	no	86.0 (111)
65	TP53:c.832C>G p.(Pro278Ala)	5	no	no	NA	73.0 (177)
72	TP53:c.711G>T p.(Met237Ile)	5	no	no	no	86.0 (89)
77	TP53:c.808_817del p.(Phe270fs)	5	no	no	NA	61.0 (81)
85	TP53:c.524G>A p.(Arg175His)	5	no	no	no	55.0 (12)
*86**	none		NA	no	NA	no
107	PIK3CA:c.277C>T p.(Arg93Trp)	4	no	NA	no	13.0 (6)
107	TP53:c.818G>A p.(Arg273His)	5	no	NA	2.4 (14)	no
*114**	ARID1A:c.2063A>G p.(His688Arg)	3	5.8 (12)	NA	no	no
124	TP53:c.672+1G>T p.?	5	no	no	no	55.0 (22)
127	ARID1A:c.2911G>A p.(Gly971Arg)	3	no	4.6 (42)	NA	no
127	PIK3CA:c.1634A>C p.(Glu545Ala)	5	no	0.24 (8)	NA	19.0 (235)
127	PIK3CA:c.1634A>G p.(Glu545Gly)	5	no	0.95 (32)	NA	no
127	TP53:c.427G>A p.(Val143Met)	5	no	no	NA	48.0 (68)
130	CTNNB1:c.121A>G p.(Thr41Ala)	5	no	no	NA	38.0 (1360)
130	PIK3CA:c.3203dup p.(Asn1068fs)	5	no	no	NA	36.0 (923)
135	ARID1A:c.4993+1G>A p.?	5	0.10 (8)	no	0.38 (6)	3.4 (6)
135	KRAS:c.37G>T p.(Gly13Cys)	5	no	no	35.0 (512)	no
135	PIK3CA:c.316G>C p.(Gly106Arg)	5	no	no	40.0 (323)	no
135	PTEN: c.968dup p.(Asn323fs)	5	no	no	80.0 (1117)	no
135	TP53:c.743G>A p.(Arg248Gln)	4	no	no	no	72.0 (186)
151	TP53:c.574C>T p.(Gln192*)	5	no	2.3 (33)	no	no

151	TP53:c.637_639delinsTGG p.(Arg213Trp)	5	no	0.18 (6)	no	90.0 (19)
160	TP53:c.743G>C p.(Arg248Pro)	5	no	no	no	71.0 (163)
*164**	None		no	no	NA	no
165	CTNNB1:c.134C>T p.(Ser45Phe)	5	no	no	no	4.5 (26)
165	MTOR:c.4448G>A p.(Cys1483Tyr)	5	no	no	no	5.5 (62)
165	PIK3CA:c.1030G>A p.(Val344Met)	5	no	no	42.0 (1668)	no
167	CTNNB1:c.110C>G p.(Ser37Cys)	5	no	no	no	27.0 (499)
167	KRAS:c.35G>T p.(Gly12Val)	5	no	no	no	39.0 (1415)
167	PIK3CA:c.323G>A p.(Arg108His)	5	no	no	no	42.0 (1668)
167	PIK3CA:c.1625A>T p.(Glu542Val)	4	no	no	9.2 (40)	no
168	ARID1A:c.4101+2T>C p.?	5	1.2 (18)	no	NA	no
168	CTNNB1:c.110C>G p.(Ser37Cys)	5	no	0.10 (7)	NA	44.0 (72)
*181**	none		no	NA	NA	no
184	PIK3CA:c.1634A>G p.(Glu545Gly)	5	0.4 (45)	0.17 (12)	4.2 (6)	no
184	TP53:c.839G>A p.(Arg280Lys)	4	no	no	no	41.0 (898)
188	TP53:c.724T>A p.(Cys242Ser)	4	no	no	no	87.0 (141)
196	PIK3CA:c.277C>T p.(Arg93Trp)	4	NA	no	NA	70.0 (1574)
196	TP53:c.747G>C p.(Arg249Ser)	5	NA	no	NA	62.0 (281)
*197**	none		NA	no	NA	no
198	KRAS:c.35G>T p.(Gly12Val)	5	NA	4.7 (34)	no	13.0 (6)
198	PIK3CA:c.277C>T p.(Arg93Trp)	4	NA	6.15 (16)	no	27.0 (75)
198	PTEN:c.955_958del p.(Thr319*)	5	NA	12.0 (465)	no	63.0 (324)
**Control patients**						
4	none		no	no	no	no
12	none		no	no	no	no
28	none		no	no	no	no
30	none		no	no	no	no
33	none		no	no	no	no
34	none		no	no	no	no
38	PIK3CA:c.1634A>G p.(Glu545Gly)	5	no	1.8 (18)	no	no
53	none		no	no	no	no
60	PIK3CA:c.1634A>C p.(Glu545Ala)	5	no	no	3.7 (22)	no
62	none		no	no	no	no
67	none		no	no	no	no
68	KRAS:c.37G>T p.(Gly13Cys)	5	no	no	no	20.0 (303)
73	none		no	no	no	no
74	KRAS:c.35G>T p.(Gly12Val)	5	no	no	no	30.0 (44)
76	none		no	no	no	no
89	none		no	no	no	no
102	none		no	no	no	no
104	none		no	no	no	no
115	none		no	no	no	no
116	none		no	no	no	no
125	none		no	no	no	no

van Bommel MH.D, *et al. Int J Gynecol Cancer* 2022; 32:1568–1575. doi: 10.1136/ijgc-2022-003911

129	none		no	no	no	no
131	none		no	no	no	no
133	none		no	no	no	no
136	none		no	no	no	no
144	none		no	no	no	no
150	ARID1A:c.1270T>C p.(Ser424Pro)	3	2.5 (20)	no	no	no
150	PIK3CA:c.3203dup p.(Asn1068fs)	5	no	no	2.5 (10)	no
153	PIK3CA:c.1634A>G p.(Glu545Gly)	5	no	2.6 (6)	no	no
159	none		no	no	no	no
166	none		no	no	no	no
183	none		no	no	no	no
195	none		NA	no	no	NA

** Excluded from analysis as sequencing of the surgical specimen was unsuccessful: coverage was too low to potentially detect any variant*
VAF, variant allele frequency; NA, not applicable; Pathogenic class: 3, variant of unknown significance; 4, likely pathogenic; 5, pathogenic

van Bommel MH.D, *et al. Int J Gynecol Cancer* 2022; 32:1568–1575. doi: 10.1136/ijgc-2022-003911

Supplementary Document 2. *Detection rate per sampling method by stage*

In table:

	All stages		Early stage (I/II)		Late stage (III/IV)	
	n	%	n	%	n	%
Self-sample	6/27	22.2	1/7	14.3	5/20	25.0
Pap smear	10/26	38.5	3/5	60.0	7/21	33.3
Pipelle	7/23	30.4	1/4	25.0	6/19	31.6
Surgical specimen	24/29	82.8	7/7	100	17/22	77.3
Total	28/29	96.6	7/7	100	21/22	95.5

In figure:

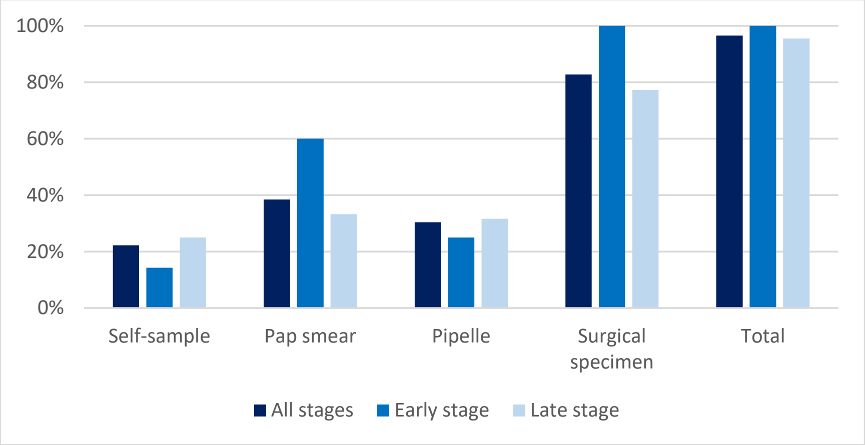

Supplement: Supplementary data [file ijgc-2022-003911supp001.pdf]
